# Troubleshooting of plastic stent impaction in the working channel during endoscopic ultrasound-guided hepaticogastrostomy

**DOI:** 10.1055/a-2836-1624

**Published:** 2026-04-15

**Authors:** Toji Murabayashi, Hirofumi Okuda, Shinya Sugimoto

**Affiliations:** 1 37071 Department of Gastroenterology, Ise Red Cross Hospital, Ise, Japan

Two previous reports 1 2 have described plastic stent impaction within the working channel during endoscopic ultrasound-guided hepaticogastrostomy (EUS-HGS) due to breakage of the thread connecting the stent to its delivery system. In both cases, troubleshooting was performed without scope exchange, resulting in stent fracture. We report successful troubleshooting of this complication by exchanging to a forward-viewing endoscope, which enabled direct visualization and improved pushability through coaxial alignment between the stent delivery system and the endoscope channel.

**Fig. 1:**
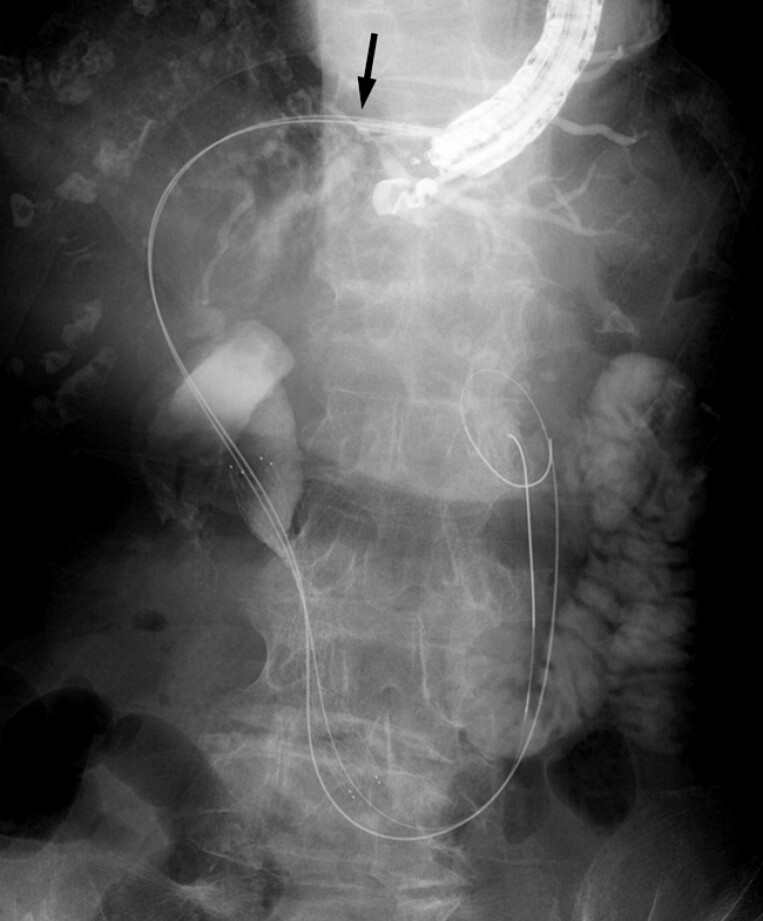
A fluoroscopic image showing a 7-Fr plastic stent impacted within the working channel after breakage of the thread connecting the stent to its delivery system, with the distal tip (arrow) embedded in the hepatic parenchyma.

**Fig. 2:**
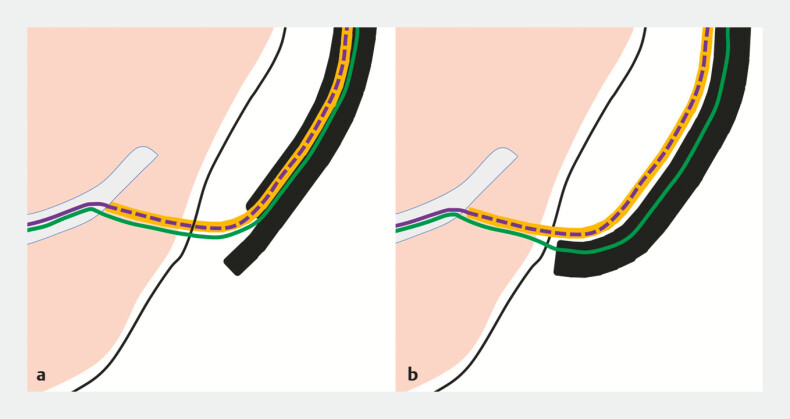
Schematic representation of the present case. **a** A 7-Fr plastic stent is impacted within the working channel, with the distal tip embedded in the hepatic parenchyma. Two guidewires remain in the bile duct. **b** A forward-viewing endoscope is positioned over the 0.025-inch guidewire within the working channel, facilitating coaxial alignment for advancement of a new plastic stent alongside the impacted stent.

**Fig. 3:**
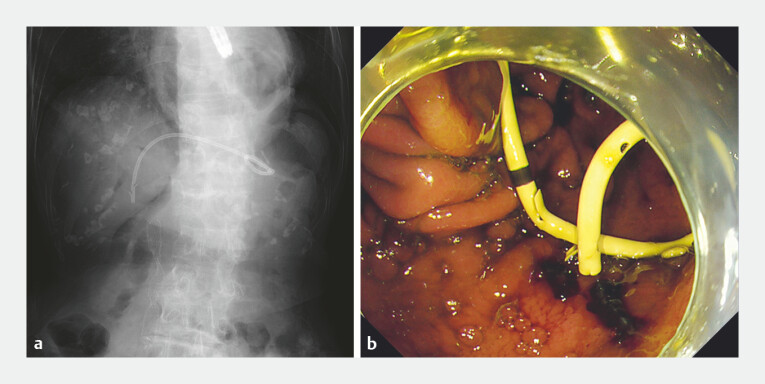
**a** Fluoroscopic and **b** endoscopic images at the completion of the procedure. **a** A 7-Fr plastic stent is successfully placed bridging the bile duct and the stomach. **b** The 7-Fr plastic stent is visible within the stomach.

An 84-year-old woman with malignant distal biliary obstruction due to pancreatic cancer underwent EUS-HGS with antegrade stenting after failed transpapillary drainage. Using a convex-array echoendoscope (GF-UCT290; Olympus Medical Systems, Tokyo, Japan), the B3 was punctured transgastrically, and two guidewires (0.025-inch and 0.035-inch) were inserted into the bile duct to establish a double-guidewire technique 3 . After antegrade stenting with a 10-mm uncovered metal stent, a 7-Fr plastic stent (Through & Pass, Type IT; Gadelius Medical, Tokyo, Japan) was advanced over the 0.035-inch guidewire but failed to traverse the bile duct wall. During attempted withdrawal, the connecting thread between the stent and the delivery system broke, resulting in stent impaction within the working channel while the distal tip remained embedded in the hepatic parenchyma ( Fig. 1 , Fig. 2 **a** ). Retrieval was attempted using a Soehendra stent retriever (Cook Medical Japan, Tokyo, Japan) but failed because the retriever tip could not engage the stent. The echoendoscope was carefully withdrawn, leaving the impacted stent and both guidewires in place ( Video 1 ). A forward-viewing endoscope (GIF-H290T; Olympus) was then advanced into the stomach over the 0.025-inch guidewire. A new 7-Fr Type IT stent was successfully advanced into the bile duct alongside the impacted stent over the same guidewire ( Fig. 2 **b** ). After deployment of the new stent, the impacted stent was grasped with forceps and removed together with the endoscope ( Fig. 3 ).

Troubleshooting of plastic stent impaction in the working channel during endoscopic ultrasound-guided hepaticogastrostomy.Video 1

Endoscopy_UCTN_Code_TTT_1AS_2AH
